# Extracellular HMGB1 as Inflammatory Mediator in the Progression of Mycoplasma Gallisepticum Infection

**DOI:** 10.3390/cells11182817

**Published:** 2022-09-09

**Authors:** Yingjie Wang, Lulu Wang, Fuli Hu, Mengyun Zou, Ronglong Luo, Yingfei Sun, Tengfei Wang, Qiao Guo, Xiuli Peng

**Affiliations:** Key Laboratory of Agricultural Animal Genetics, Breeding and Reproduction, Ministry of Education, College of Animal Science and Technology and College of Veterinary Medicine, Huazhong Agricultural University, Wuhan 430070, China

**Keywords:** HMGB1, TLR2/TLR4, *Mycoplasma gallisepticum*, NF-κB signaling pathway, inflammatory cytokine storm

## Abstract

High-mobility group box 1 (HMGB1), a member of damage-associated molecular patterns (DAMPs), is involved in the immune regulation of several infectious diseases. *Mycoplasma gallisepticum* (MG) infection is proved to cause an abnormal immune response, but the role of HMGB1 in MG-induced chronic respiratory disease (CRD) is unclear. In this study, we found that HMGB1 was released from the nucleus to the extracellular in macrophages upon infection with MG. Extracellular HMGB1 bound to TLR2 activating the NF-κB pathway triggering a severe inflammatory storm and promoting the progression of MG infection. More importantly, TLR4 could be activated by HMGB1 to trigger immune disorders after TLR2 was silenced. This disease process could be interrupted by ethyl pyruvate (EP) inhibition of HMGB1 release or glycyrrhizic acid (GA). Furthermore, treatment of MG-infected chickens with GA significantly alleviated immune organ damage. In conclusion, we demonstrate that HMGB1 is secreted extracellularly to form an inflammatory environment upon MG infection, triggering a further cellular inflammatory storm in a positive feedback approach. Blocking MG-induced HMGB1 release or suppression downstream of the HMGB1-TLR2/TLR4 axis may be a promising novel strategy for the treatment of CRD. Furthermore, this study may provide a theoretical reference for understanding non-LPS-activated TLR4 events.

## 1. Introduction

Damage-associated molecular patterns (DAMPs) are endogenous molecules that activate the immune system by interacting with pattern recognition receptors (PRRs) such as Toll-like receptors (TLRs), RIG-I-like receptors (RLRs), NOD-like receptors (NLRs), and non-pattern recognition receptors [1,2]. High-mobility group box 1 (HMGB1), typical of DAMPs, is a nuclear protein that is commonly expressed and highly conserved in almost all eukaryotic cells with multiple roles in a variety of pathophysiological processes [3]. It can be transferred from the nucleus to cytoplasm and extracellular compartments to perform immune functions upon exposure to cellular stress [4]. HMGB1 is released both through active secretion or passively following various types of cell death (such as necrosis, apoptosis, pyroptosis, and ferroptosis) and serves as an inflammatory mediator to induce cellular immune responses [5]. Extracellular HMGB1 may bind to soluble extracellular immune activating molecules or directly to cell surface receptors of immune cells (mainly TLR2 and TLR4) [6]. It further propagates intracellular cascade signals that promote the expression and release of inflammatory cytokines, mediating/triggering inflammation and activating innate and adaptive immunity [7,8].

TLR2 and TLR4 are key TLRs types that closely relate to HMGB1. HMGB1 stimulation upregulates TLR2 and TLR4 levels in macrophages [9]. It has been shown that the mechanism of HMGB1-induced inflammation and injury is associated with TLR4/MD-2. TLR4/MD-2 mediates macrophage activation and overproduction of cytokine release [10]. HMGB1 itself possesses the ability to induce TLR4 signaling, presenting a new idea for the study of TLR4 pathway activation by non-LPS or non-TLR4 ligands [11]. Numerous studies have shown that HMGB1 activates the IKK kinase complex, including IKKα and IKKβ, through TLR4 receptor signaling, which then leads to phosphorylation of IκB and nuclear translocation of NF-κB [12]. The persistent activation of the NF-κB pathway leads to sustainable cellular inflammation and triggers immune disorders [13].

A key characteristic of pathogenic *mycoplasma* that interferes with the host response is its ability to alter the secretion of immunomodulatory substances [14]. *Mycoplasma gallisepticum* (MG), a major pathogenic microorganism causing chronic respiratory disease (CRD) in chickens, persistently triggers the release of immune factors after infection of the host to maintain chronic inflammation [15,16,17]. HMGB1 activates TLRs receptors and is extensively involved in the infection process of pathogens. Previous studies have shown that MG can promote the expression of HMGB1 [18]. Another study shows that MG neither produces LPS nor contains TLR4 ligands but activates the TLR4 pathway [19]. Therefore, we explored the mechanism of MG infection based on the hypothesis that MG may activate the TLR4 signaling pathway by altering the expression of HMGB1.

In this study, we investigated the mechanism of HMGB1 release during MG infection and its role in triggering immune disorders. Our results provide a new perspective on the treatment of MG-induced CRD. More importantly, it will facilitate theoretical reference for comprehension of HMGB1 as a broad-spectrum anti-infectious pathogen therapeutic target.

## 2. Materials and Methods

### 2.1. Mycoplasma Strains

MG-HS, a virulent strain, was reported in detail in our previous studies [20]. MG-HS was presented by the State Key Laboratory of Agricultural Microbiology, College of Veterinary Medicine, Huazhong Agricultural University (Wuhan, China). The MG-HS strain was inoculated in modified FM-4 full-valent medium supplemented with 12% of activated pig serum and cultured in a biochemical incubator (temperature of 37 °C, humidity at 56%) until the phenol changed from red to orange in the medium. The concentration of viable *Mycoplasmas* in a suspension was then determined by a color-changing unit (CCU) assay [21]. In this study, the concentration of MG-HS used was 1 × 10^12^ CCU/mL.

### 2.2. HD-11 Cells Culture and Treatment

HD-11 cells, chicken macrophage-like cell lines transformed by avian myelocytoma virus (MC 29), were presented by Professor Zhuang Ding of Jilin University. Dulbecco’s Modified Eagle Medium (DMEM) (Gibco, Shanghai, China) containing 10% fetal bovine serum (FBS) was used to culture HD-11 cells at 37 °C and 5% CO_2_ concentration.

To investigate the effect of HMGB1 release on the progression of MG infection, the 6 h post-infection cells were treated with glycyrrhizic acid (GA) (purity ≥ 98%, Luoyang, Henan, China) or ethyl pyruvate (EP) (purity ≥ 98%, Sigma, St. Louis, MO, USA). Specifically, HD-11 cells were inoculated in 6-well plates at 1 × 10^5^ cells/well. Once the cell density reached 60–70% confluence, cells were challenged with MG-HS (50 μL, 1 × 10^12^ CCU/mL) and after 4 h, they were treated with GA (50 μg/mL) or PE (50 μg/mL) for 24 h.

### 2.3. Mycoplasma Gallisepticum Quantification

To determine the extent of MG infection, a standard curve was established using a cloned MG gene recombinant plasmid to detect the absolute abundance of MG by quantitative real time PCR [22]. Briefly, DNA from cells or spleens were extracted under aseptic conditions and the 16Sribosomal RNA gene of MG was subsequently amplified as part of the MG genomic DNA using qPCR and sequenced. In addition, two-step PCR thermal cycling for DNA amplification and real-time data acquisition were performed with an ABI StepOnePlus™ Real-Time PCR System using the following cycle conditions: 95 °C for 1 min × 1 cycle, and 95 °C for 15 s, followed by 60 °C for 1 min × 40 cycles. Fluorescence data were analyzed by the ABI StepOnePlus software and expressed as CT; the number of cycles needed to generate a fluorescent signal above a predefined threshold. The sequences of 16SrRNA primers are shown in the Table 1.

### 2.4. Gene Overexpression and Knockdown Assays

The CDS fragment of the HMGB1 gene was cloned into the pcDNA3.1 vector to construct the HMGB1 overexpression plasmid (marked as End-HMGB1). HMGB1 recombinant protein was purchased from Abcam (Marked as Exo-HMGB1). Small interfering RNA (siRNA) of genes were all synthesized by GenePharma (Suzhou, China) and all RNA oligonucleotides sequences are shown in Table 2. Once 80–90% confluence was achieved, cells were transfected with End-HMGB1 to increase the expression of endogenous HMGB1 or supplemented with Exo-HMGB1 to enhance the levels of extracellular HMGB1. In addition, HD-11 cells were transfected with Si-HMGB1, Si-TLR2, or Si-TLR4 to knock down the expression of each gene and were named HMGB1-KD, TLR2-KD, and TLR4-KD, respectively.

### 2.5. Immunofluorescent Staining

After fixation in 4% paraformaldehyde, cells were permeabilized using 0.1% Triton X-100 and then blocked with 1% BSA. Furthermore, 200 μL Rabbit anti-NF-κB p65 primary antibody (at 1:200 dilution) (Cell Signaling Technology, MA, USA) were incubated overnight at 4 °C, and were then incubated with 200 μL fluorescently labeled secondary antibodies (at 1:1000 dilution) for 1 h at 37 °C. Nuclei were stained with DAPI for 5 min. Finally, fluorescence confocal microscopy was used to observe cellular localization of p65.

### 2.6. Protein Extraction and Western Blot

Twenty-four hours after infection, the total proteins were extracted from HD-11 by lysis buffer (formulated at a ratio of 99 to 1 of RIPA to PMSF). Nucleus Protein Extraction Kits (#A10008, Abmart, Shanghai, China) and Cytoplasm and Membrane Extraction Kits (#A10009, Abmart, Shanghai, China) were used to extract nucleus, cytoplasm, and membrane proteins according to the manufacturer’s instructions. Then, the protein concentrations were determined using a Bicinchoninic acid (BCA) protein assay reagent kit (Transgen, Shanghai, China). Then, 12% SDS-polyacrylamide gel electrophoresis (Beyotime, China) was used to separate equal amounts of protein, followed by blocking with 5% BSA for 1 h. Then, primary antibodies for HMGB1 (Abmart, M001355), Tubulin (Abmart, M20005), YY1 (Abcam, ab227269), TLR-4 (Affinity Biosciences, AF7017), Caspase3(Abmart, T4046), Bcl-2 (ABmart, A19693), Bcl-XL (ABmart, A0209), Caspase9 (Abmart, 40046), IκB-α (Abmart, T55026), p-IκB-α (Abmart, TP56290), p65 (Abclonal, A11201), p-p65 (Abcam, ab76302) (all at 1:2000 dilution), and GAPDH (Abmart, M20025) or β-actin (Abmart, T40104) (at 1:5000 dilution) proteins were incubated overnight at 4 °C. Finally, the membrane was incubated with a secondary antibody for 1 h after TBST washing. The enhanced chemiluminescence (ECL) detection system (Bio-Rad) was used to detect the protein expression.

### 2.7. ELISA

HD-11 cells were inoculated on a 12-well plate at 5 × 10^6^ cells per well. Twenty-four hours after treatment, the supernatants were collected and the HMGB1 levels were detected with enzyme-linked immunosorbent assay kits (Meimian, Jiangsu, China, MM-168201) according to the manufacturer’s directions.

### 2.8. RNA Isolation and Quantitative Real-Time PCR

According to the manufacturer’s instructions, total mRNA was isolated from post-infected and non-infected cells via TRNzol Universal Reagent kit (TIANGEN, Beijing, China). Then, RNA was inverse transcribed to cDNA with the first strand cDNA systhesis kit (TaKaRa, Tokyo, Japan) and reverse transcription PCR (RT-PCR).

### 2.9. Experimental Animals and Treatments

One hundred and twenty chickens free of specific pathogenic bacteria were kept for one week to adapt to the experimental conditions before the test. Ad libitum feed and water were provided to chickens. These chickens were housed in the experimental chicken farm of Huazhong Agricultural University. After one week, the chickens were randomly divided into 4 experimental groups of 3 replicates each, with 10 chickens in each replicate. Experimental groups included control group (marked as blank); GA alone treated group (100 mg/kg) (marked as blank); infection groups challenged with MG-HS (marked as MG); infection group treated with GA (100 mg/kg) (marked as MG + GA). Challenge and treatment methods were detailed in previous publications [23]. One week after therapy, chickens were humanely executed (30 chickens per group) to avoid suffering and spleen tissues were collected for further experimental analysis.

### 2.10. Histopathological Analysis of Spleen

Fresh spleen tissues were collected from each group, then trimmed into small pieces of appropriate size and fixed in 10% neutral formalin at room temperature for more than 48 h. Then, they were paraffin-embedded, gradient alcohol dehydrated, and hematoxylin and eosin stained (H&E). The pathological changes of tissue were observed by optical electron microscopy.

### 2.11. Terminal Deoxynucleotidyl Transferase—Mediated dUTP Nick Endlabeling Assay

Terminal deoxynucleotidyl transferase-mediated dUTP nick end labeling (TUNEL) assay was used to identify apoptotic cells in chicken spleen as described earlier [15]. In brief, formalin-fixed tissues were dehydrated in standard ethanol and then embedded in paraffin. Tissue sections were stained according to the manufacturer’s instructions using the TUNEL assay kit (Elabscience, Wuhan, China) and, subsequently, fluorescent microscopy was used to detect apoptotic cells. Apoptotic cell rate was calculated as number of apoptotic cells/number of total cells × 100%.

### 2.12. Statistics

Data and results are presented as means ± SD. Student’s t-test was used to determine significant differences between groups. A value of *p* < 0.05 was considered statistically significant and >0.05 was considered non-significant. (* *p* < 0.05, ** *p* < 0.01, NS *p* > 0.05.). All the graphs were made by GraphPad prism software (Created by Harvey Motulsky, window version 8.0.2, San Diego, CA, USA).

## 3. Results

### 3.1. MG Infection Induces the Secretion of HMGB1

**Figure 1:**
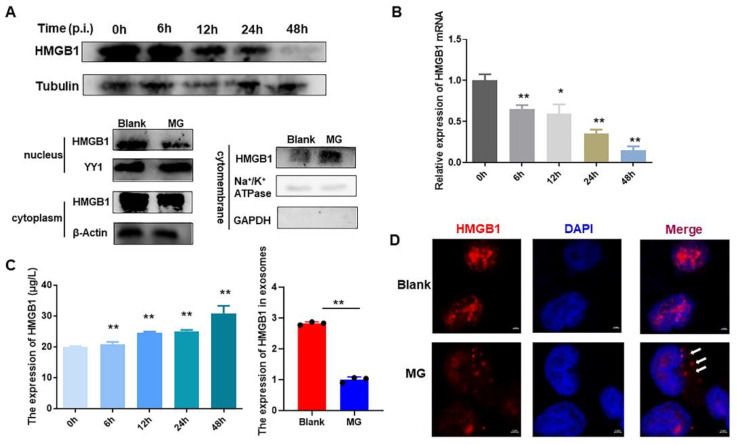
MG infection induces the secretion of HMGB1 in HD-11 cells. (**A**) The protein expression of HMGB1 was detected by WB. Tubulin, YY1, β-actin, or Na+/K+ ATPase are considered as the loading control. In addition, GAPDH was shown as the negative control of membrane protein. (**B**) The mRNA expression of HMGB1 HD-11 cells. GAPDH was used for normalization (n = 4). (**C**) Left: HMGB1 levels in the supernatant were measured by HMGB1 Elisa kit (n = 4). Right: The expression of HMGB1 was detected in the exosomes derived from MG-infected cells (n = 3). (**D**) Confocal images of HMGB1 (red) and DAPI (bule) in HD-11 cells. White arrows: membrane-localized HMGB1 (scale bar, 2 µm). * *p* < 0.05 and ** *p* < 0.01 show statistically significant difference compared to the 0 h group. Black dots in bar graph represent datasets from independent experiments.

To investigate the role performed by HMGB1 in MG infection, its expression was first examined in MG-infected HD-11 cells. The results showed that HMGB1 levels were downregulated with the progression of MG infection (Figure 1A,B). Twenty-four hours after infection, nucleus, plasma, and membrane of HD-11 were isolated and HMGB1 expression was assayed individually in these cellular fractions. HMGB1 is decreased in the nucleus and cytoplasm but enriched in the cell membrane in MG-infected cells (Figure 1A). Meanwhile, the HMGB1 content of cell supernatant was measured at different times after MG infection. We found that HMGB1 levels in cell supernatants were time-dependently upregulated by MG infection. Interestingly, HMGB1 was not enriched in exosomes derived from MG-infected cells (Figure 1C). Moreover, immunofluorescence results also demonstrated that MG affected the cellular localization of HMGB1 (Figure 1D).

### 3.2. Extracellular HMGB1 Affects MG Replication

**Figure 2:**
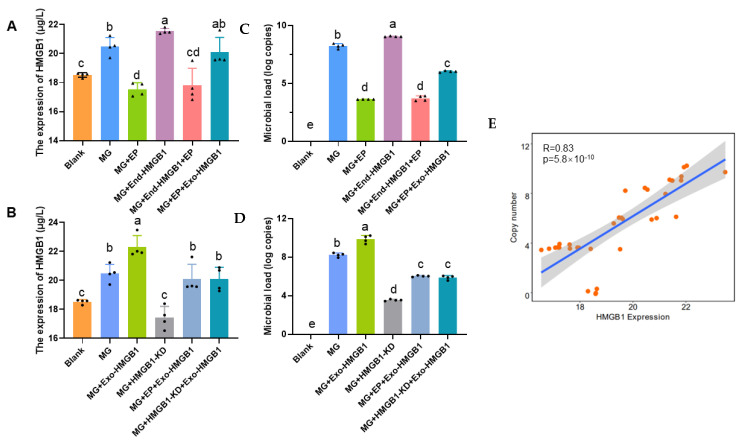
HMGB1 positive correlation with MG load. HD-11 cells were infected with MG for 24 h and cell supernatants (**A**,**B**) and cells were collected (**C**,**D**). The HMGB1 levels were detected by ELISA. Meanwhile, the quantity of MG was detected. EP, the HMGB1 release inhibitor; End-HMGB1, the overexpression plasmid of HMGB1; Exo-HMGB1, the HMGB1 recombinant protein. (**E**) A correlation chart between extracellular HMGB1 and MG load, their correlation degree was R = 0.83. Different lowercase letters represent *p* < 0.05. Black dots in bar graph represent datasets from independent experiments.

To explore the effect of HMGB1 release on MG infection, HD-11 cells overexpressed or knocked down HMGB1 to assess the proliferation of MG. Cell supernatants and cells were collected 24 h after treatment of MG-infected cells with HMGB1 release inhibitor (EP, a high security HMGB1 release inhibitor [24]) and HMGB1 recombinant protein (Exo-HMGB1). We found that extracellular HMGB1 increased by endogenously increasing HMGB1 expression or by supplementing it with exogenous HMGB1, while knocking down the HMGB1 gene or EP reduced its levels. Even though the HMGB1 gene was overexpressed, there was no increase in HMGB1 concentration in the cell supernatant due to the specific release inhibitory effect of EP. Meanwhile, knockdown or release inhibition of HMGB1 could be rescued by Exo-HMGB1(Figure 2A–D). Subsequently, the load of MG was examined, and we found a positive correlation between extracellular HMGB1 and microbial load (Figure 2E).

### 3.3. Extracellular HMGB1 Activates TLR2/4 to Promote MG Proliferation

**Figure 3:**
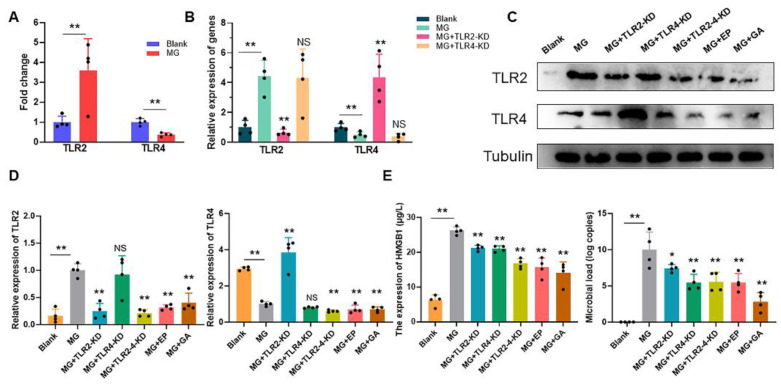
HMGB1 promotes MG infection via TLRs. (**A**–**D**) HD-11 cells were infected with MG for 24 h and cells were collected. The TLRs levels were detected by qPCR or WB. (**E**) **Left**: HMGB1 levels in the supernatant were measured by HMGB1 Elisa kit. **Right**: The MG colonization in HD-11 cells was detected. The data are the mean ± S.D. of at least four independent experiments. NS *p* > 0.05, * *p* < 0.05, and ** *p* < 0.01 show statistically significant difference compared to the MG group. Black dots in bar graph represent datasets from independent experiments.

TLR2 and TLR4 as receptors for HMGB1 participate in immune responses in macrophages [25]. MG infection significantly upregulated TLR2 but suppressed TLR4 expression (Figure 3A). TLR2 or TLR4 in MG-infected HD-11 cells were knocked down by using siRNA and detecting their expression (Figure 3B). We subsequently further explored the targeting relationship between extracellular HMGB1 and TLRs. HMGB1 first activated TLR2 receptors and, thus, promoted MG replication but not TLR4. Upon deletion of TLR2, TLR4 exhibited a compensatory effect activated by HMGB1 (Figure 3C). More importantly, either TLR2-4 co-knockdown, EP administration or GA (a direct HMGB1 receptor antagonist [26]) treatment reduced TLR2-4 expression and inhibited MG proliferation (Figure 3C,D).

### 3.4. HMGB1-Activated TLR2-4 Triggers Immune Disorders via NF-κB

**Figure 4:**
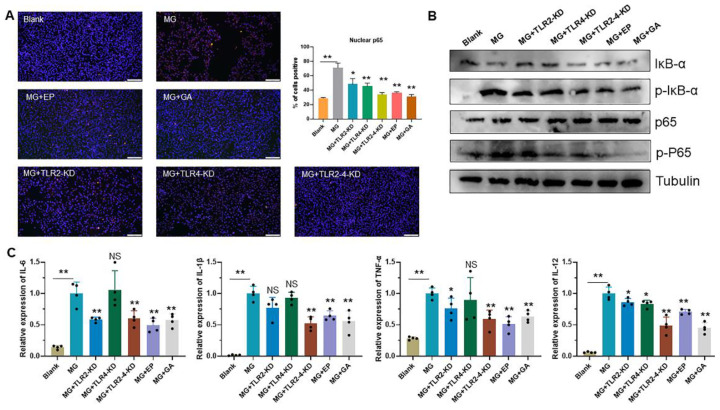
HMGB1 activates the NF-κB pathway via TLRs. (**A**) Translocation of the NF-κB p65 subunit from the cytoplasm into the nucleus was assessed by immunofluorescence staining. NF-κB p65 was stained with red; cell nucleus was stained blue with DAPI (scale bar, 100 µm). (**B**) The protein expression of NF-κB pathway-related genes in different treatment groups were detected by Western blotting. GAPDH was used for normalization. (**C**)The relative expression levels of TNF-α, IL-1β, IL-12, and IL-6 mRNA in HD-11 cells. NS *p* > 0.05, * *p* < 0.05, and ** *p* < 0.01 show statistically significant difference compared to the MG group. Black dots in bar graph represent datasets from independent experiments.

**Figure 5:**
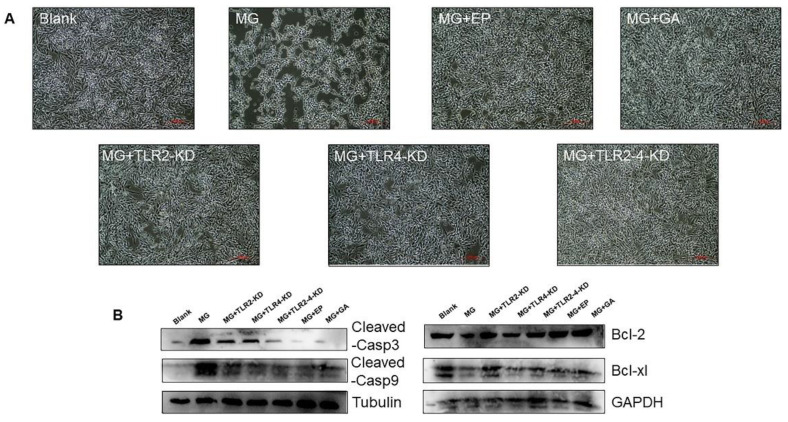
Inhibition of HMGB1 alleviates MG-induced apoptosis. (**A**) HD-11 cells apoptosis observed by microscopy (scale bar, 200 µm). (**B**) The proteins of apoptosis genes were detected by Western blotting. Tubulin and GAPDH were used for normalization.

TLRs act as recognition molecules for the immune response and activate the NF-κB pathway to participate in immune regulation [27]. Here, we demonstrated that MG activated the NF-κB pathway, increased the phosphorylation levels of p65 and IκB-α and promoted p65 entry into the nucleus. This process could be alleviated by applying EP or GA or knocking down TLRs (Figure 4A,B). In addition, we found that NF-KB activation provoked a severe immune factor storm. Pro-inflammatory factors including IL-6, IL-1β, IL-12, and TNF-α dramatically upregulated after secretion of HMGB1 by MG infection. As expected, EP, GA, and TLR2-4 deletion treatments all significantly attenuated the cytokine storm. However, the alleviation of immune disorders by knocking down TLR2 or TLR4 alone remained unimpressive due to the HMGB1 preferential recognition effect of TLR2 and the compensation effect of TLR4 (Figure 4). The immune system overreaction damages itself. We also confirmed that TLR2-4 co-deletion, EP and GA-mitigated immune factor storms suppressed MG-induced cell death (Figure 5A). MG promoted the expression of pro-apoptotic genes but suppressed the level of apoptotic genes. This phenomenon is reversed upon blocking HMGB1 binding to receptor (Figure 5B).

### 3.5. Disruption of HMGB1 Binding to TLR2-4 Receptors In Vivo Alleviates MG-Induced Immune Organ Damage

**Figure 6:**
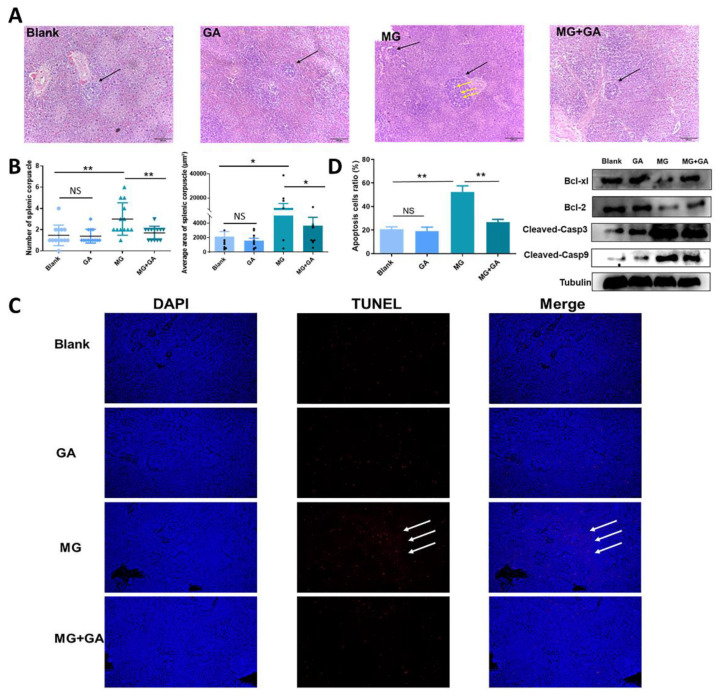
Neutralization of HMGB1 alleviates MG-induced splenic damage. (**A**) Spleen tissue sec-tion (H & E staining). Orange arrows shows lymphocytes shedding and splenic lymph nodes were significantly enlarged. Splenic corpuscle (black arrows). (**B**) The number of splenic corpuscles and the mean area of splenic corpuscle. (**C**) TUNEL assay kit was used to detect the extent of apoptosis in the spleen tissue. White spots (scale = 50 µm) indicate positive TUNEL staining (apoptotic cells). Bar graph shows mean results ± SD (n = 3). (**D**) Left: Quantification of the degree of spleen tissue apoptosis by fluorescence intensity. Right: The proteins of apoptosis genes were detected by Western blotting. Tubulin was used for normalization. NS *p* > 0.05. * *p* < 0.05, and ** *p* < 0.01 show statistically significant difference compared to the MG group. Black dots in bar graph represent datasets from independent experiments.

The above results have demonstrated that MG induced HMGB1 release to facilitate infection, while blocking HMGB1 release or inhibiting HMGB1 downstream were both effective in alleviating disease progression. To further verify them, GA was performed in vivo to inhibit the binding of HMGB1 to TLR2-4. Histopathological sections were used to assess pathological changes in the immune organ spleen. A typical histopathological change such as a marked decrease in lymphocytes and a significant upregulation in the number and average size of splenic nodules was observed in the MG-infected groups. These histopathological changes were evidently alleviated by GA (100 mg/kg) (Figure 6A,B). The spleen apoptosis was evaluated by TUNEL assay. As shown in Figure 6, GA treatment significantly reduced the rate of apoptosis in the MG-infected spleen compared to the infected group (a reduction of approximately 30%). In addition, MG activated the expression of casp3 and casp9 but inhibited bcl2 and bcl-xl in spleen tissue, which was consistent with in vitro experiments (Figure 6C,D).

**Figure 7:**
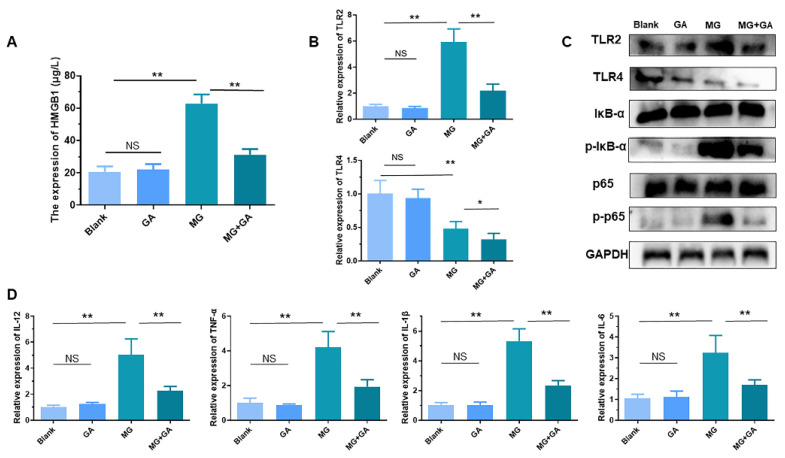
Inhibition of HMGB1 alleviates MG-induced inflammatory damage in spleen tissue. (**A**) The level of HMGB1 in serum was detected by ELISA. (**B**,**C**) The levels of TLRs and NF-κB related-genes were detected by qPCR and WB. (**D**) The relative expression levels of TNF-α, IL-1β, IL-12, and IL-6 mRNA in the spleen tissue. NS *p* > 0.05. * *p* < 0.05, and ** *p* < 0.01 show statistically significant difference compared to the MG group.

Expectedly, MG infection increased the level of HMGB1 in chicken serum compared to the uninfected groups (Figure 7A). GA treatment significantly moderated serum HMGB1 concentration. Similar to experiments in vitro, TLRs-NFκB pathway genes were examined. Infection with MG increased TLR2 levels and thereby activated the NF-κB pathway. GA decreased TLR2/4 levels in MG-infected spleen (Figure 7B). GA reduced phosphorylation, but not levels, of p65 and IκB-α proteins (Figure 7C). More importantly, GA interruption of NF-κB activation alleviated MG-HMGB1-triggered over-release of immune factors (Figure 7D).

## 4. Discussion

Damage-associated molecular patterns (DAMPs) are endogenous molecules that activate the immune system and are associated with infectious diseases [28]. HMGB1, a typical molecule of DAMPs, normally resides in the nucleus [29]. Upon exposure to infectious agents or endogenous danger signals, including bacteria, viruses, endotoxin (LPS), and extracellular adenosine triphosphate (ATP), immune cells can translocate HMGB1 nuclei into the cytoplasm or even extracellularly [8,30]. HMGB1 with different subcellular localization possesses diverse biological functions [31]. In the present study, we demonstrated that HMGB1 level in HD-11 cells after MG infection was downregulated as MG infection progressed (Figure 1A,B). MG affected the subcellular localization of HMGB1 and promoted the release of HMGB1 (Figure 1D). More importantly, we found that exosomes derived from MG-infected cells were not enriched for HMGB1. These results predicted that MG-induced release of HMGB1 was not dependent on the exosome pathway (Figure 1C). It has been shown that apart from exosomal pathway release, HMGB1 can be dependent on the release of the gasdermins family [32], live cells can release HMGB1 via the lysosomal pathway [4,33], and another study showed that the rupture of the cell membrane causes HMGB1 release [4]. However, the mechanism of MG-induced HMGB1 translocation to the extracellular space is unclear and further investigation is required.

Numerous studies have shown that HMGB1 participates in the replication and infection process of pathogens [34]. Intracellular HMGB1 functions as a proviral factor that promotes hepatitis C virus (HCV) replication in hepatocytes by interacting with the HCV genome [35,36]. HMGB1 protein binds to influenza virus nucleoprotein (NP) in nucleus and affects viral replication by maintaining viral polymerase activity [37]. It also inhibits the activity and function of immune cells and exacerbates LPS-induced acute lung injury (ALI) [38]. These results suggest that HMGB1, whether in cytoplasm or in the nucleus, can directly or indirectly regulate the progression of infection by pathogens. However, the intracellular presence of HMGB1 does not always facilitate pathogen replication. In this study, we determined that intracellular HMGB1 was not affected by MG replication through overexpression or inhibition of endogenous HMGB1 levels. The extracellular HMGB1, however, was positively correlated with the load of MG (Figure 2). Our results were in line with other studies in showing that extracellular, but not intracellular, HMGB1 affects Newcastle disease virus (NDV) or porcine reproductive and respiratory syndrome virus (PRRSV) levels [39,40].

In addition, it has been reported that the serum HMGB1 level in HCV-infected patients with liver cancer is significantly higher than that in healthy individuals [41]. The concentration of HMGB1 in serum is positively correlated with tumourigenesis and metastasis [42,43]. It is, therefore, considered to be a pathological parameter for determining cancer progression after surgery. Similar to cancer patients, greater amounts of HMGB1 are released into the serum of patients with influenza A virus infection, which is associated with severe pneumonia [44,45]. Furthermore, a dramatic upregulation of HMGB1 was observed in the sera of mice exposed to LPS-induced ALI [46]. These studies support the fact that HMGB1 released in the serum following pathogen infection is pathogenic. In the current study, we found MG-infected chickens contained high levels of HMGB1, which reaffirmed the pathogenicity of secretory HMGB1 (Figure 1C and Figure 7A).

It is possible that blocking the release of HMGB1 may be a viable broad-spectrum anti-disease strategy as HMGB1 protein is released during infection by most pathogens [47]. Inhibition of dengue virus infection by the chemical resveratrol through blocking the nuclear to cytoplasmic transport of HMGB1, leading to the accumulation of HMGB1 in the nucleus [48]. Intravenous administration of HMGB1-neutralising monoclonal antibody significantly improves survival in H1N1/H5N1-infected mice [49,50]. HCV infection in Huh7 cells is reduced by treatment with HMGB1 inhibitor GA, and the nucleus-cytoplasmic translocation of HMGB1 induced by viral infection is blocked [51,52]. It has also been shown that paeonol can treat LPS-induced ALI in rats by inhibiting the expression, translocation, and secretion of HMGB1 [53]. These results demonstrate that either the blockade of HMGB1 release or the antibody neutralization response can mitigate the progression of pathogenic infections. In this study, we explored the role of HMGB1 in the model of MG-induced CRD. It was demonstrated that MG infection triggered the release of HMGB1 and accelerated its infection progression. Consistent with previous studies of different pathogens, both inhibition of HMGB1 release or repression of HMGB1 downstream receptors were able to suppress MG replication (Figure 2).

Toll-like receptors (TLRs) recognize exogenous pathogen-associated molecular patterns (PAMPs) and endogenous danger-associated molecular patterns (DAMPs) and initiate the immune response [2]. Extracellular HMGB1 stimulates immune cells such as macrophages/monocytes, dendritic cells, and eosinophils through the activation of a series of signaling cascades including MAPK and NF-κB, which is essential for the induction of the release of cytokines such as IL-8, IL-6, and TNF-α [54,55]. TLR2/TLR4 participate in a variety of immune regulation as typical HMGB1 receptors [56]. The immune system eliminates pathogens by secreting massive amounts of cytokines [57]. However, secretory HMGB1 exerts excessive activation of immune cells, induces overexpression of pro-inflammatory cytokines, and triggers immune disorders [58]. For example, LPS induces the release of HMGB1 to activate TLR2/TLR4 to cause inflammatory storm [59]. A study on the pathogenesis of ALI reveals that HMGB1 activates protein kinase R (PKR) in macrophages through TLR2- and TLR4-mediated NF-κB signaling pathways, inducing M1 polarization [60]. The release of HMGB1 triggered by PRRSV infection increases the efficiency of virus-induced inflammatory damage through the overproduction of inflammatory cytokines by RAGE, TLR2, and TLR4 [40,61]. In addition, a study on the MG-R_low_ strain showed that MG-R_low_ infection upregulated HMGB1 expression and activated the TLR4 signaling pathway, causing severe lung injury and intestinal flora imbalance in chickens. These results implicate the possibility that HMGB1 activates the TLRs cascade response in the MG-HS infection process. In this study, we, therefore, first verified the relationship between HMGB1 and TLR2/4. We identified that after MG infection HMGB1 first activated TLR2, but not TLR4, triggering an immune response (Figure 3A). In contrast, upon TLR2 deficiency, TLR4 appeared to exhibit a compensatory effect in response to MG infection (Figure 3B,C). This phenomenon corresponded to TLR2-deficient mice treated with transverse aortic constriction (TAC) [62]. In addition, our results showed that TLR2/4 activated by HMGB1 further activates the NF-κB pathway to produce immune disorders (Figure 3D, Figure 4A,B, and Figure 7B,C). An excessive release of inflammatory factors, including IL-6, IL-12, IL-1β, and TNF-α, from immune disorders caused severe apoptotic damage to immune organs (Figure 4C and Figure 7D), which was consistent with MG-R_low_ infection-induced damage to the spleen, thymus, and bursa of fasciola [63,64]. More importantly, knockout of the TLR2/4 receptor of HMGB1 or inhibition of its binding to the receptor were able to significantly interrupt the immune disruption cascade stimulated by HMGB1. These approaches all attenuated excessive inflammation-caused macrophage apoptosis and immune organ (spleen) damage (Figure 5 and Figure 6). It indicates that apart from interrupting the release of HMGB1, measures to block its binding to the receptor are also a potentially viable anti-disease strategy.

## 5. Conclusions

**Figure 8:**
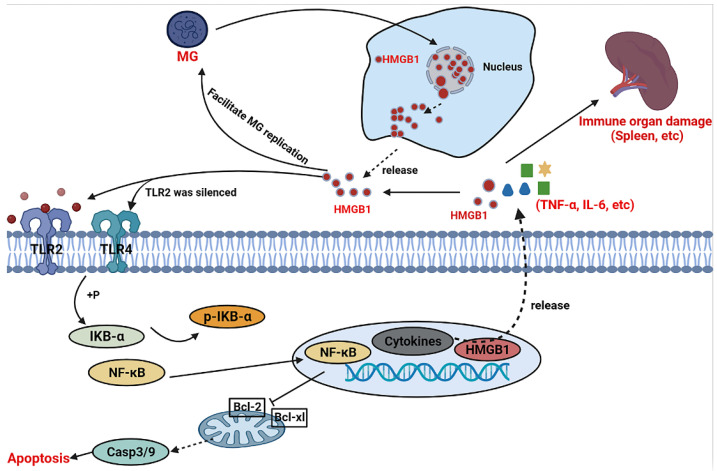
Schematic diagram. MG invasion of immune cells (such as chicken macrophages, HD-11) increases the level of inflammatory mediators (such as HMGB1) in the extracellular environment and, thus, worsens the progression of infection. In vivo, enrichment of inflammatory mediators in immune organs (e.g., spleen) aggravates their inflammatory damage.

In conclusion, our study demonstrates that MG infection induces the release of HMGB1 in a non-exosomal approach to activate TLR2/4 and participate in immune regulation. HMGB1-induced immune disorder provokes inflammatory storms causing cell and organ damage via the TLR2/4-NF-κB signaling pathway (Figure 8). Removal of MG-induced extracellular HMGB1 or inhibition of HMGB1 downstream may be a promising novel strategy for the treatment of CRD. Furthermore, our results provide a novel theoretical reference for comprehension of HMGB1 aggravating pathogenic infections and pathogenesis.

## Figures and Tables

**Table 1:** Sequences of DNA primers.

Name	Primer Sequence (5′-3′)
	Primers for CDS Cloning
HMGB1-CDS-F	CCGCTCGAGCGGATGGGCAAAGGTGATCCCAA
HMGB1-CDS-R	CCGGAATTCCGGTTATTCATCATCATCATCATCTTCC
	Primers for RT-qPCR
GAPDH-F	GAGGGTAGTGAAGGCTGCTG
GAPDH-R	CACAACACGGTTGCTGTATC
TLR-2-F	CGCAAGCTTATGTTCAACCAAAG
TLR-2-R	CGCCTCGAGCTATGACTTCAAGG
TLR-4-F	ATCTTTCAAGGTGCCACATC
TLR-4-R	GGATATGCTTGTTTCCACCA
TNF-α-F	GGACAGCCTATGCCAACAAG
TNF-α-R	ACACGACAGCCAAGTCAACG
IL-1β-F	ACTGGGCATCAAGGGCTACA
IL-1β-R	GCTGTCCAGGCGGTAGAAGA
IL-12-F	TGGAACGATGAGACACCAGC
IL-12-R	AGACAGGCAGGTGTAGTTGC
IL-6-F	CTCCTCGCCAATCTGAAGTC
IL-6-R	CCCTCACGGTCTTCTCCATA
16 SrRNA-F	AGCTAATCTGTAAAGTTGGTC
16SrRNA-R	CGCTTCCTTGCGGTTAGCAAC
HMGB1-F	AAGGTGATCCCAAGAAGCCG
HMGB1-R	GAAGCTTGTCAGCCTTTGCC

**Table 2:** Sequences of RNA oligonucleotides.

Name	Sequences (5′-3′)
Si-TLR2	GCCAUGCAAACUUUCACAATT
	UUGUGAAAGUUUGCAUGGCTT
Si-TLR4	GCAGCCUUCCAUGGCUUAATT
	UUAAGCCAUGGAAGGCUGCTT
Si-HMGB1	GCAGAUGAUAAACAGCCUUTT
	AAGGCUGUUUAUCAUCUGCTT
NC	UUCUCCGAACGUGUCACGUTT
	ACGUGACACGUUCGGAGAATT

## Data Availability

The data that support the findings of this study are available from the corresponding author, Xiuli Peng, upon reasonable request.
